# Prognostic Value of the PILE Score in Esophageal Squamous Cell Carcinoma Treated with Neoadjuvant Chemoradiotherapy

**DOI:** 10.3390/diagnostics15243158

**Published:** 2025-12-11

**Authors:** Aykut Turhan, Mehmet Emin Büyükbayram, Zekeriya Hannarici, Alperen Akansel Çağlar, Mehmet Bilici, Salim Başol Tekin

**Affiliations:** 1 Department of Medical Oncology, Ordu University Training and Research Hospital, Ordu 52200, Turkey; 2 Department of Medical Oncology, Yalova Training and Research Hospital, Yalova 77200, Turkey; 3 Department of Medical Oncology, Bursa Yüksek İhtisas Training and Research Hospital, Bursa 16310, Turkey; 4 Department of Medical Oncology, Istanbul Çam and Sakura City Hospital, Istanbul 34480, Turkey; 5 Department of Medical Oncology, Atatürk University Faculty of Medicine, Erzurum 25040, Turkey

**Keywords:** esophageal squamous cell carcinoma, neoadjuvant chemoradiotherapy, PILE score, overall survival, progression-free survival

## Abstract

**Background:** This retrospective cohort study evaluated the role of the PILE score as a prognostic biomarker for overall survival (OS) and progression-free survival (PFS) in patients with locally advanced esophageal squamous cell carcinoma (ESCC) who were treated with neoadjuvant chemoradiotherapy (nCRT). **Methods:** This study included 108 patients with ESCC treated with weekly paclitaxel-carboplatin and concurrent radiotherapy at Erzurum Atatürk University Faculty of Medicine Hospital between January 2018 and April 2024. Patients were categorized into low- (PILE score 0–1) and high-risk (PILE score 2–3) groups. Kaplan–Meier analysis and Cox regression models were used to evaluate the association between the PILE score and survival outcomes. **Results:** The results showed that the high-risk PILE group had significantly shorter median OS (18.6 months vs. not reached; *p* < 0.001) and PFS (12.4 months vs. not reached; *p* < 0.001) than the low-risk group. Multivariate analysis showed that the PILE risk classification [hazard ratio (HR) = 2.527; 95% confidence interval (CI): 1.380–4.629; *p* = 0.003] and surgical resection (HR = 0.249; 95% CI: 0.090–0.683; *p* = 0.007) were independent prognostic factors for OS, whereas the PILE risk classification (HR = 2.932; 95% CI: 1.525–5.639; *p* = 0.001) and surgical resection (HR = 0.131; 95% CI: 0.044–0.394; *p* < 0.001) were independent prognostic factors for PFS. **Conclusions:** The study concludes that the PILE score is a robust prognostic tool for OS and PFS in patients with ESCC undergoing nCRT, highlighting its potential for risk stratification and personalized treatment planning.

## 1. Introduction

Esophageal cancer is a significant global health concern and is among the leading causes of cancer-related deaths [1,2]. In 2020, there were an estimated 604,100 cases of this cancer, with 544,100 deaths, and this number is expected to increase [1]. Certain regions, particularly East Asia and Africa, experience high incidence and mortality rates, forming “the esophageal cancer belt” [3,4]. Within this belt, esophageal squamous cell carcinomas account for the vast majority of all esophageal cancer cases, often exceeding 90% [5,6]. Despite advancements in treatment methods, the prognosis remains poor for cases diagnosed at an advanced stage, highlighting the urgent need for enhanced strategies and techniques [7].

Currently, nCRT followed by esophagectomy is regarded as the standard approach for treating locally advanced resectable esophageal cancers, including ESCC [8,9]. The landmark CROSS trial provided strong evidence for the effectiveness of nCRT protocols that involve weekly doses of paclitaxel and carboplatin in combination with radiation, showing significant enhancements in overall survival and R0 resection rates, particularly in the ESCC group, compared to those who underwent only surgery [9,10]. Subsequent studies have reinforced the effectiveness and tolerability of paclitaxel-carboplatin regimens for nCRT [11,12].

Beyond traditional clinicopathological factors, systemic inflammation has emerged as a significant prognostic indicator in a range of cancers, including ESCC [13]. Blood tests, such as the neutrophil-to-lymphocyte ratio and platelet-to-lymphocyte ratio, are recognized as biomarkers that reflect the host’s inflammatory response to tumors, influencing both tumor progression and treatment response [14,15,16]. Additionally, the Prognostic Inflammatory Index, which combines various inflammatory markers, is valuable for predicting patient outcomes in different cancers [17,18].

While certain inflammatory biomarkers offer valuable prognostic insights, their incorporation into more intricate scoring systems can enhance prognostic precision. The PILE score exemplifies this combined prognostic tool by integrating the Pan-Immune-Inflammation Value (PIV), lactate dehydrogenase (LDH) level, and Eastern Cooperative Oncology Group (ECOG) performance status [19]. Elevated LDH levels are indicative of a substantial tumor burden and aggressive disease, which correlates with poor prognostic outcomes [20,21]. Conversely, ECOG performance status represents the clinician’s subjective assessment of a patient’s overall functional status and is recognized as an independent prognostic factor of overall survival [19]. Despite the prognostic utility of the PILE score in other cancers, such as extensive-stage small-cell lung cancer, particularly concerning immunotherapy treatment, its prognostic value in the context of ESCC and nCRT remains underexplored [19]. There is a need to validate the PILE score in specific populations and contexts [22].

This study aimed to explore the prognostic value of the PILE score for OS and PFS in patients with locally advanced ESCC who underwent neoadjuvant chemoradiotherapy.

## 2. Materials and Methods

### 2.1. Study Design and Setting

**Figure 1:**
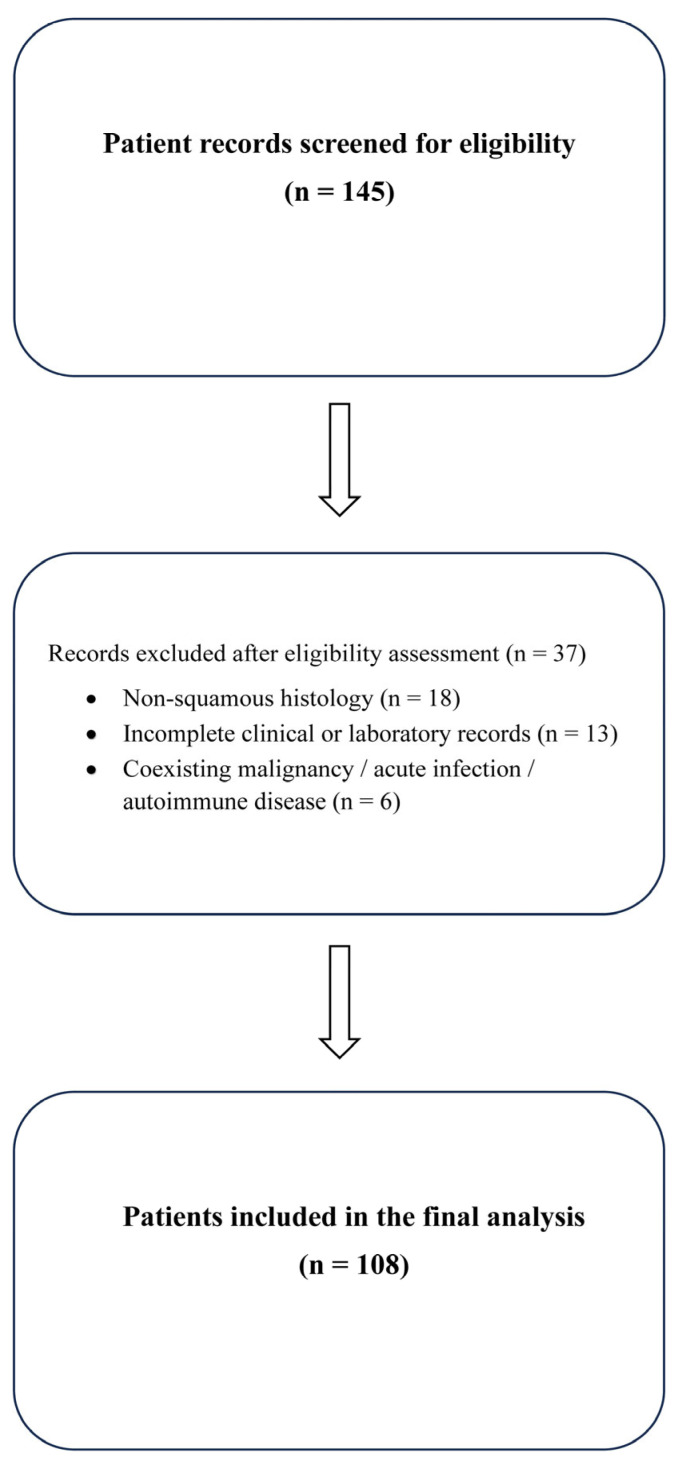
Flow diagram of patient selection: A total of 145 patients were screened for eligibility. After exclusions for non-squamous histology (*n* = 18), incomplete clinical or laboratory records (*n* = 13), and coexisting malignancy, acute infection, or autoimmune inflammatory conditions (*n* = 6), 108 patients were included in the final analysis.

This retrospective cohort study was conducted at the Erzurum Atatürk University Faculty of Medicine Hospital. This study included patients diagnosed with ESCC through histological examination, all of whom underwent nCRT between January 2018 and April 2024 at our hospital. Participants were required to be ≥18 years of age and have clinical stage II–IVA ESCC. The exclusion criteria were incomplete medical records, missing initial laboratory data, non-squamous histology, the presence of another active cancer, active infections, or known autoimmune or chronic inflammatory diseases, to prevent confounding of systemic inflammatory markers. A total of 145 patients were screened. Of these, 18 were excluded due to non-squamous histology, 13 due to incomplete medical or laboratory records, and 6 due to coexisting malignancy, acute infection, or autoimmune inflammatory conditions. The remaining 108 patients comprised the final study cohort. A detailed flowchart of patient selection is presented in Figure 1.

### 2.2. Treatment Protocol

All patients received a weekly regimen of neoadjuvant chemoradiotherapy based on paclitaxel and carboplatin. Chemotherapy involved administering paclitaxel at a dose of 50 mg/m^2^ and carboplatin with an area under the curve (AUC) of 2, administered weekly over five to six cycles. External beam radiotherapy was also administered, totaling 41.4 to 50.4 Gray (Gy), divided into 23 to 28 fractions, with each fraction being 1.8 to 2.0 Gy. Following the completion of nCRT, the patients were re-evaluated, and surgical resection was performed only for those considered suitable by the multidisciplinary tumor board.

### 2.3. Data Collection

Data on demographic, clinical, pathological, and treatment-related factors, including age, sex, smoking habits, existing health conditions, ECOG performance status, tumor site, clinical T/N stage, overall clinical stage, surgical status, and disease progression, were extracted from electronic hospital records. Laboratory values, such as neutrophil, lymphocyte, monocyte, platelet, and LDH levels, were measured within seven days before starting nCRT.

### 2.4. Inflammatory Indexes and PILE Score

The neutrophil-to-lymphocyte ratio (NLR) was calculated by dividing the absolute neutrophil count by the lymphocyte count, and the platelet-to-lymphocyte ratio (PLR) was calculated by dividing the platelet count by the lymphocyte count. The PIV was determined using the following formula: (neutrophils × platelets × monocytes)/lymphocytes. The PILE score comprises three factors assessed together: PIV of 413 or higher (median threshold), LDH levels exceeding the upper normal limit (ULN), and ECOG performance status of 2 or more, with each factor adding 1 point. At our institution, the ULN for LDH is 240 U/L, and values above this threshold are considered elevated. The PILE score ranges from 0 to 3, categorizing patients into low- (0–1 points) or high-risk (2–3 points) groups. A cohort-specific median PIV cut-off (413) was selected, which is consistent with previous studies on lymphoma, hepatocellular carcinoma, and small-cell lung cancer, where median-based thresholds were used owing to the absence of universally validated cut-offs for inflammation-based markers [19,23,24].

### 2.5. Outcome Definitions

OS was measured as the duration from the time of diagnosis to death from any cause or until the most recent follow-up. PFS was defined as the period from treatment initiation to radiologically confirmed disease progression, recurrence or death.

### 2.6. Statistical Analysis

Continuous variables are expressed as mean ± standard deviation (SD), and categorical variables are expressed as counts and percentages. To compare the discriminative performance of the individual inflammatory markers (NLR, PLR, and PIV) with the composite PILE score, receiver operating characteristic (ROC) curve analysis was performed, and the AUC values were calculated. The Kaplan–Meier method was used to estimate survival outcomes, and comparisons were performed using the log-rank test. Univariate Cox proportional hazards analyses were conducted to identify possible prognostic factors for OS and PFS. Variables identified in the univariate analysis were included in the multivariate Cox regression model, and the results are presented as HRs and 95% CIs. Both variables with *p* < 0.10 in the univariate analysis and clinically relevant covariates were included in the multivariate model. To avoid collinearity, ECOG performance status and LDH level were excluded from the multivariate model once the composite PILE score was introduced, as these variables were already embedded within it. Therefore, the multivariate analysis assessed the prognostic effect of this composite index rather than its individual components. Model performance was assessed using the concordance index (C-index). The association between the PILE score and pathological complete response (pCR) was examined using Pearson Chi-square and Fisher’s exact test. Statistical analyses were performed using IBM SPSS Statistics version 31.0 (IBM Corp., Armonk, NY, USA), and a *p*-value of less than 0.05 was deemed statistically significant.

Additionally, patients were classified into two treatment cohorts: those who underwent esophagectomy after neoadjuvant chemoradiotherapy and those treated with definitive chemoradiotherapy (dCRT), defined as chemoradiation without subsequent surgery. Predefined subgroup Cox regression analyses were performed to evaluate whether the prognostic effect of the PILE score differed between the treatment groups. An interaction term (PILE × surgical status) was incorporated into the multivariable Cox regression models for both OS and PFS to formally assess the effect modification.

### 2.7. Language Editing Statement

AI-assisted language editing tools [Paperpal (version 4.4.1) and ChatGPT (OpenAI GPT-5.1, 2025 release)] were used only for grammar correction, paraphrasing, and translation support during the final proofreading stage.

## 3. Results

### 3.1. Patient Characteristics

This study analyzed 108 patients with ESCC who underwent neoadjuvant chemoradiotherapy consisting of weekly paclitaxel (50 mg/m^2^) and carboplatin (AUC 2) for five to six cycles, delivered concurrently with external beam radiotherapy totaling 41.4–50.4 Gy in 23–28 fractions (1.8–2.0 Gy per fraction). The average age of the participants was 62.3 ± 10.8 years (range: 37–89 years). The group comprised 63.9% females and 36.1% males. The majority of patients (66.7%) were non-smokers, 29.6% were current smokers, and 3.7% had quit smoking. The ECOG performance statuses were 0 (25.9%), 1 (53.7%), and 2 (20.4%), respectively. Comorbid conditions were found in 47.2% of the patients, with hypertension (20.4%), coronary artery disease (10.2%), diabetes mellitus (9.3%), and chronic obstructive pulmonary disease (COPD) (2.8%) being the most common. The tumors were primarily located in the lower third of the esophagus (75.0%), with the remaining 25.0% located in the middle third. Clinically, 37.0% of the patients had T2 tumors, whereas 63.0% had T3 tumors. Nodal involvement was categorized as N0 in 24.1%, N1 in 32.4%, N2 in 32.4%, and N3 in 11.1% of patients, corresponding to clinical stages II (37.0%), III (51.9%), and IVA (11.1%) disease. Post-neoadjuvant therapy and surgical resection were performed in 31.5% of patients. Among the surgically treated patients, pCR was achieved in 25 of 34 patients (73.5%). During follow-up, 39.8% of the patients experienced disease progression, and 14 patients developed distant metastases, primarily to the lungs (50%) and liver (21.4%). At the last follow-up, 57.4% of patients were alive. Laboratory tests showed a mean LDH level of 216.8 ± 51.5 U/L, lymphocyte count of 2.17 ± 0.68 × 10^9^/L, neutrophil count of 5.35 ± 2.01 × 10^9^/L, monocyte count of 0.61 ± 0.19 × 10^9^/L, and platelet count of 296.4 ± 101.1 × 10^9^/L. The mean PIV was 550.0 ± 577.6 (median PIV: 413), whereas the mean NLR and PLR values were 2.75 ± 1.68 (0.81–12.90) and 148.4 ± 66.9 (59.6–435.3), respectively. According to the PILE scoring system, 70.4% of patients were classified as low-risk (0–1), whereas 29.6% were considered high-risk (2–3). The mean OS was 26.34 ± 15.32 months and the mean PFS was 22.01 ± 15.39 months. The details of the study are summarized in Table 1.

### 3.2. ROC Analysis for Discriminatory Performance

To assess the prognostic capabilities of the PILE score against traditional inflammatory markers (NLR, PLR, and PIV), ROC curve analysis was conducted. The PILE score exhibited a higher ability to distinguish survival outcomes, achieving an AUC of 0.709, which surpassed that of PLR (0.631), NLR (0.572), and PIV (0.567) scores. When the PILE risk classification was categorized into low- and high-risk groups, the AUC increased to 0.715, demonstrating strong prognostic differentiation. These findings validate that the composite PILE score offers significantly enhanced prognostic accuracy compared with individual inflammatory markers.

### 3.3. Overall Survival Analysis

Kaplan–Meier analysis revealed no statistically significant difference in OS between males and females, with median OS of 41.4 months for males and 54.6 months for females (*p* = 0.336). The ECOG performance status has emerged as a significant prognostic factor for survival. Patients with an ECOG score of 0–1 experienced notably longer OS, whereas those with an ECOG score of 2 had significantly shorter survival times (median OS: ECOG 0 = 45.9 months, ECOG 1 = 54.6 months, ECOG 2 = 16.1 months; *p* = 0.006).

The presence of comorbidities did not lead to a notable difference in overall survival (median OS: no comorbidity = not reached, comorbidity present = 33.7 months; *p* = 0.463). Although survival rates appeared to decline with higher clinical stages, this variation was not statistically significant (median OS: stage II, 45.9 months; stage III, not reached; stage IVA, 18.6 months; *p* = 0.100).

Surgical resection is closely associated with enhanced OS. Patients who underwent surgery had a notably longer lifespan than those who did not undergo the procedure (median OS: 24.7 months for non-surgical patients versus not reached for surgical patients; *p* < 0.001).

**Figure 2:**
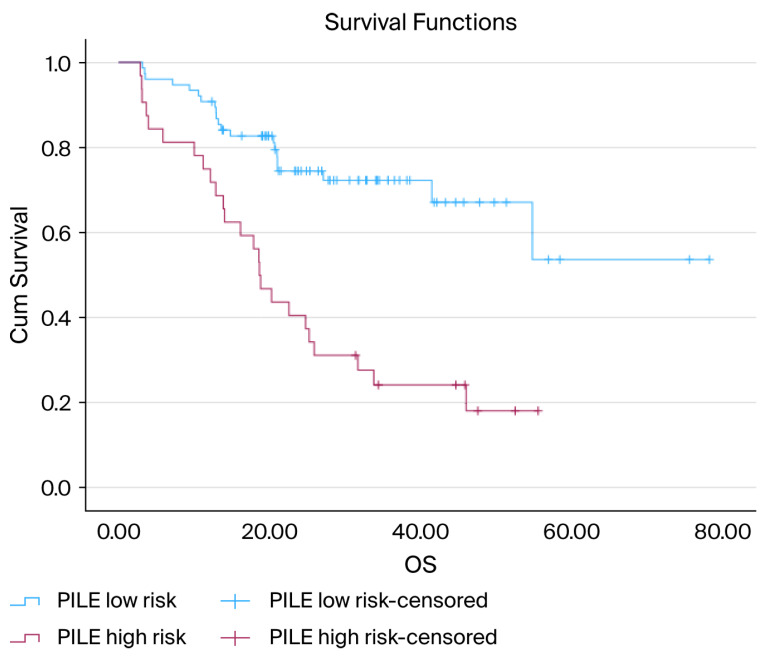
Kaplan–Meier curves for OS according to the PILE risk groups: Kaplan–Meier OS curves stratified by the PILE risk classification. Patients in the low-risk group (PILE score 0–1) experienced significantly longer OS than those in the high-risk group (PILE score 2–3). The median OS was not reached in the low-risk group, but was 18.6 months in the high-risk group. The difference between the survival curves was statistically significant (log-rank, *p* < 0.001).

The PILE risk classification was the most effective in distinguishing OS outcomes. Patients classified as low-risk had a notably longer survival period, whereas those in the high-risk category experienced significantly worse OS (median OS: low-risk = not reached, high-risk = 18.6 months; *p* < 0.001) (Figure 2).

### 3.4. Cox Regression Analysis for OS

In the univariate Cox regression analysis, age was significantly correlated with the OS hazard ratio (HR = 1.032; 95% CI: 1.005–1.060; *p* = 0.021). However, sex, comorbidity status, and clinical stage were not significantly associated with overall survival (*p* = 0.338, 0.464, and 0.111, respectively). Nonetheless, in the categorical analysis of clinical stage, patients with stage IVa disease had a significantly higher risk than those with stage II disease (HR = 2.38; 95% CI: 1.02–5.52; *p* = 0.044). Surgical resection emerged as a strong protective factor, with patients who underwent surgery exhibiting notably improved survival rates (HR = 0.181; 95% CI: 0.071–0.460; *p* < 0.001). Additionally, PILE risk classification was significantly associated with OS, with high-risk patients experiencing considerably poorer survival outcomes (HR = 3.531; 95% CI: 1.974–6.318; *p* < 0.001).

In the multivariate Cox regression analysis, PILE risk classification (HR = 2.527; 95% CI: 1.380–4.629; *p* = 0.003) and surgical resection (HR = 0.249; 95% CI: 0.090–0.683; *p* = 0.007) were independent prognostic factors for OS. Factors such as age, sex, comorbidities, and clinical stage were not statistically significant in the adjusted model (*p* > 0.05). The C-index of the model was 0.469, reflecting a moderate discriminatory power (Table 2).

#### Subgroup and Interaction Analysis for OS

Subgroup analyses were conducted to assess whether the prognostic significance of the PILE score varied between patients who underwent surgery and those who received dCRT. In the group that did not undergo surgery, the PILE score emerged as a robust and significant prognostic factor for overall survival (HR = 2.64; 95% CI: 1.42–4.91; *p* = 0.002). Conversely, in the group of patients who underwent surgery, the association between the PILE score and overall survival was not statistically significant (HR = 2.62; 95% CI: 0.41–16.81; *p* = 0.310), likely due to the small sample size and limited statistical power.

The interaction term between the PILE score and surgical status was non-significant (HR = 1.44; 95% CI: 0.22–9.62; *p* = 0.707), suggesting that the prognostic impact of the PILE score was not notably different across the two groups.

### 3.5. Progression-Free Survival Analysis

According to the Kaplan–Meier analysis, there was no notable difference in PFS between men and women, with the median PFS not being reached for men and standing at 27.3 months for women (*p* = 0.357). Patients without comorbid conditions experienced a longer PFS than those with comorbidities, with the median PFS not reached versus 24.7 months, respectively (*p* = 0.031).

ECOG performance status significantly influenced PFS. Patients with an ECOG score of 0–1 experienced notably longer PFS, whereas those with an ECOG score of 2 had the shortest survival duration (median PFS: ECOG 0 = not reached, ECOG 1 = not reached, ECOG 2 = 10.2 months; *p* < 0.001).

Although the clinical stage appeared to be associated with a trend toward reduced PFS as the disease progressed, this variation did not reach statistical significance (median PFS: stage II, 40.8 months; stage III, not reached; stage IVA, 19.8 months; *p* = 0.311).

Surgical resection was strongly associated with enhanced PFS. Patients who underwent surgery experienced a notably longer PFS than those who did not, with the median PFS not being reached versus 18.2 months (*p* < 0.001).

**Figure 3:**
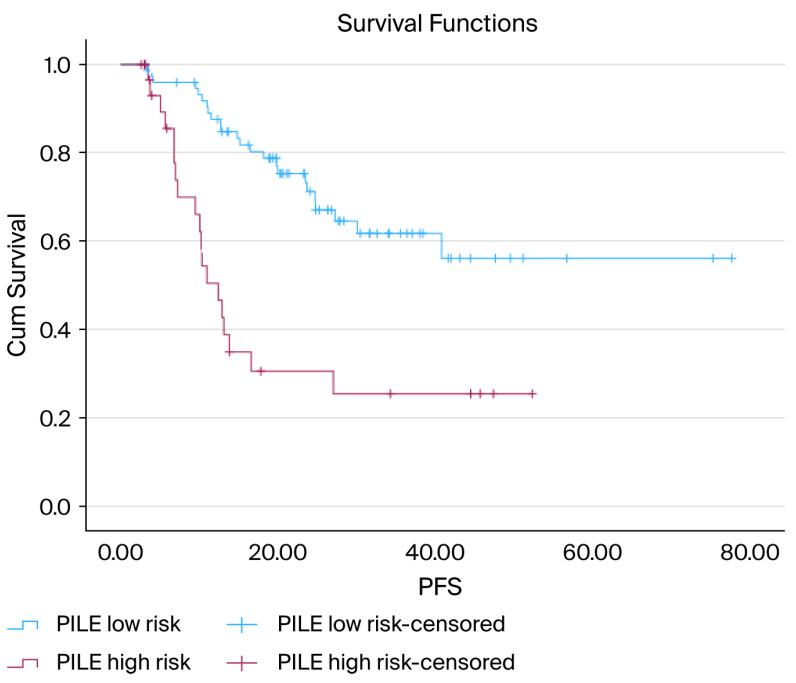
Kaplan–Meier curves for PFS according to PILE risk groups: Kaplan–Meier PFS curves comparing low-risk (PILE score 0–1) and high-risk (PILE score 2–3) groups. The low-risk group demonstrated a markedly longer PFS, whereas the high-risk group showed substantially earlier progression. The median PFS was not reached in the low-risk group and was 12.4 months in the high-risk group. The difference in survival between the groups was statistically significant (log-rank, *p* < 0.001).

The PILE risk classification effectively distinguished between the different PFS outcomes. Patients classified as low-risk had notably longer PFS, whereas those in the high-risk category had significantly worse outcomes (median PFS not reached vs. 12.4 months; *p* < 0.001) (Figure 3).

### 3.6. Cox Regression Analysis for PFS

In the univariate Cox regression analysis, age was significantly associated with reduced PFS, with an HR of 1.030 (95% CI: 1.002–1.060; *p* = 0.035). Sex was not associated with PFS (HR = 1.366; 95% CI: 0.701–2.663; *p* = 0.359). The presence of comorbidities significantly increased the risk of disease progression (HR = 1.938; 95% CI: 1.049–3.579; *p* = 0.034). Clinical stage was not significantly associated with PFS (*p* = 0.312). Surgical resection emerged as a significant protective factor, and patients who underwent surgery had significantly improved PFS (HR = 0.116; 95% CI: 0.041–0.328; *p* < 0.001). The PILE risk classification showed a strong prognostic value, as high-risk patients had notably shorter PFS (HR = 3.561; 95% CI: 1.936–6.551; *p* < 0.001).

In the multivariate Cox analysis, surgical resection and PILE risk classification were independent prognostic factors for PFS. Patients who underwent surgery had a significantly lower risk of disease progression (HR = 0.131; 95% CI: 0.044–0.394; *p* < 0.001). A high PILE risk was still associated with worse PFS outcomes (HR = 2.932; 95% CI: 1.525–5.639; *p* = 0.001). Factors such as age, sex, comorbidity status, and clinical stage were not significant in the adjusted model (*p* > 0.05). The model exhibited a strong discriminatory ability with a C-index of 0.786 (Table 3).

#### Subgroup and Interaction Analysis for PFS

Subgroup analyses were performed to assess whether the prognostic impact of the PILE score on PFS differed between patients who underwent surgery and those who received dCRT. In the group that did not undergo surgery, the PILE score emerged as a robust and statistically significant prognostic indicator of PFS (HR = 2.50, 95% CI: 1.31–4.76; *p* = 0.005). For patients who underwent surgical treatment, the PILE score also showed a significant correlation with PFS (HR = 7.30, 95% CI: 1.02–52.07; *p* = 0.047), although the broad confidence interval indicated the small sample size and reduced statistical power of this subgroup analysis.

The interaction term between the PILE score and surgical status was not statistically significant (HR = 3.15, 95% CI: 0.40–24.82; *p* = 0.275), suggesting that the prognostic impact of the PILE score on PFS was similar for patients who underwent surgery and those who underwent dCRT.

### 3.7. Association Between PILE Score and pCR

Of the 34 patients who underwent surgery, 25 (73.5%) achieved pCR. In the low PILE-risk category, 22 of 30 patients (73.3%) reached pCR, whereas in the high-risk category, three of four patients (75.0%) did so. Both the Pearson’s chi-square and Fisher’s exact tests indicated no significant difference in pCR rates between the two PILE risk categories (*p* = 0.943 and *p* = 1.000, respectively).

### 3.8. Model Performance

The ability of the Cox regression models to differentiate was assessed using the C-index. For OS, the multivariate model showed limited discrimination, with a C-index of 0.469, indicating a restricted capacity to differentiate between patients with varying survival risks. Conversely, the PFS model exhibited a strong discriminatory capability, achieving a C-index of 0.786, which signifies excellent prognostic accuracy for progression risk.

## 4. Discussion

In this study, we demonstrated that the PILE score serves as a robust and significant prognostic indicator for both overall survival and progression-free survival in patients with esophageal squamous cell carcinoma who underwent nCRT. The critical influence of the PILE score on patient outcomes, along with the substantial variation in survival rates among patients who underwent surgery after nCRT based on their PILE score, highlights the importance of the PILE score in forecasting outcomes for this challenging patient group.

Our findings align with those of earlier studies highlighting the prognostic significance of individual inflammatory biomarkers and performance status across various cancers, including ESCC. For instance, pan-immune inflammation is recognized as a prognostic factor for survival in ESCC and other solid tumors [17,18,19]. Additionally, elevated lactate dehydrogenase levels are strongly associated with worse outcomes in patients with ESCC receiving different treatments [20,21]. The ECOG performance status is a well-established prognostic biomarker that indicates a patient’s overall health and functional status [19,25]. Furthermore, studies have validated the prognostic value of other systemic inflammation markers, such as the neutrophil-to-lymphocyte and platelet-to-lymphocyte ratios, as prognostic factors for the survival of patients with ESCC undergoing concurrent chemoradiotherapy [25,26]. Although numerous studies have examined other components or combinations, such as the Gustave Roussy Immune Score [27] or CALLY Index [28], the PILE score integrates all three elements: PIV, LDH, and ECOG. To our knowledge, this is the first study to thoroughly evaluate the PILE score in patients with ESCC undergoing nCRT, thereby addressing a significant gap in understanding the role of comprehensive biomarker prognostic tools in this clinical context.

The prognostic significance of the PILE score is derived from the interconnected roles of the component biomarkers in tumor progression. A high PIV index indicates an increased level of systemic inflammation, characterized by elevated neutrophil, platelet, and monocyte counts along with reduced lymphocyte [29]. Neutrophils contribute to tumor growth, angiogenesis, and invasion by releasing factors that promote tumors, and their adaptability allows them to support tumors within the tumor microenvironment [30,31,32,33]. Platelets play a role in protecting against immune destruction and facilitating metastasis by aiding extravasation and microembolus formation, as well as contributing to tumor vascular integrity and immune evasion [34,35,36,37,38,39,40]. Monocytes transform into tumor-associated macrophages, promoting tumor growth, angiogenesis, invasion, and metastasis, which are strongly linked to poor patient outcomes [41,42,43,44,45,46,47,48,49,50]. Conversely, low lymphocyte levels are associated with an ineffective antitumor immune response, allowing tumor escape and correlating with reduced overall survival [29,51,52,53,54]. Elevated LDH levels often indicate a high tumor burden, anaerobic glycolysis, and hypoxia, all of which are associated with aggressive tumor types, resistance, and poor prognosis [20,21,55,56]. Lastly, a poor ECOG Performance Status reflects diminished general physical functioning and health, often indicating a negative prognosis and serving as a measure of functional status in patients with cancer [19,57,58,59,60,61]. By integrating these distinct yet complementary biomarkers, the PILE score enhances prognostic accuracy by providing a comprehensive view of the interaction between the host and tumor, as well as metabolic stress. Consistent with this biological rationale, our ROC analysis showed that the PILE score demonstrated superior prognostic discrimination compared with individual inflammatory markers such as NLR, PLR, and PIV, further supporting the advantage of composite inflammation-based indices over single-ratio biomarkers in predicting prognosis.

The PILE score plays a vital role in the clinical management of patients receiving nCRT. It is an effective prognostic factor for both OS and PFS, making it valuable for risk stratification in the context of nCRT. Patients with ESCC identified as high-risk by the PILE score may require special attention and follow-up, potentially involving frequent evaluations through imaging or endoscopic procedures. Furthermore, these high-risk patients with ESCC might benefit from alternative treatment strategies, such as exploring novel agents in combination with surgery as an adjunctive treatment or participating in clinical trials assessing the effectiveness of immune checkpoint inhibitors and targeted therapies, which could enhance their prognosis. The PILE score can be a significant tool in team discussions, aiding in tailoring and improving treatment decisions for physicians and, consequently, their patients. Moreover, the prognostic performance of the model was markedly stronger for PFS than for OS, as demonstrated by the substantially higher C-index (0.786 vs. 0.469), indicating that the PILE score more accurately discriminated the progression risk than the long-term mortality risk.

Surgical removal after nCRT continues to be a fundamental component of curative treatment for locally advanced ESCC, with strong evidence indicating notable enhancements in both overall survival and progression-free survival compared to nCRT alone or initial surgery [62,63,64,65]. The crucial CROSS trial and subsequent research have conclusively demonstrated the survival benefit of esophagectomy following nCRT in this group [66]. Our research further revealed that prognostic variations based on the PILE score remained evident even among patients who underwent surgical removal, highlighting its significant prognostic value. A high PILE score likely indicates not only aggressive tumor characteristics but also increased systemic inflammation and diminished physiological capacity, which may hinder the ability to endure major surgery and lead to postoperative complications and poorer long-term outcomes [67,68,69]. Moreover, an inadequate pathological response to nCRT, often linked to elevated inflammatory markers, may further worsen the surgical outcomes [70,71,72]. Consistent with its primarily prognostic nature, the PILE score did not show a significant association with pCR to nCRT in our cohort. Consequently, the PILE score could serve as a crucial perioperative risk assessment tool, assisting multidisciplinary teams in evaluating surgical eligibility, enhancing patient counseling, and identifying patients who might benefit from intensified preoperative preparation or alternative treatment approaches to minimize surgical risk and boost overall survival [73,74].

The present study had several strengths. The chief among them is that all participants were administered an identical nCRT regimen, specifically paclitaxel-carboplatin, on a weekly basis, which minimizes potential biases stemming from different nCRT treatments. Furthermore, this study leveraged real-world data to enhance the applicability of our findings to actual clinical environments. Notably, this is the first study to assess the prognostic significance of PILE in a cohort of patients with ESCC post-nCRT, marking the first effort to illuminate this complex and challenging scenario. However, this study had some limitations. The primary concern is the inherent risk of selection and unspecified biases typical of retrospective analyses. Additionally, the dataset was somewhat constrained in size, as the study was conducted at a single center, which may limit its generalizability and necessitate validation in other contexts. Inaccuracies may also exist in our PIV and LDH measurements because their levels can fluctuate under different conditions. The decision to perform elective surgery following nCRT may also be influenced by factors beyond response rates, potentially leading to inconsistencies in management practices. Another important limitation is the heterogeneity between patients who underwent surgery after nCRT and those who received dCRT. These groups differ biologically and prognostically, and although subgroup analyses were performed, residual confounding related to treatment selection could not be fully excluded. Furthermore, the use of a median PIV cutoff, rather than an optimized threshold derived from ROC analysis using the Youden Index or X-tile software, may limit the generalizability of our findings. Although median-based thresholds are widely used in inflammation-based indices, external validation in larger, independent cohorts is needed to establish a more robust and universally applicable cutoff.

### Clinical Implications and Future Research

The findings of this study indicate that the PILE score holds significant clinical value, serving not only as a prognostic biomarker before treatment but also as a potential instrument for tailoring personalized treatment plans in ESCC. By combining the PILE score with established prognostic indicators, such as tumor regression grade, pathological lymph node status, and new markers such as circulating tumor DNA, risk stratification can be improved, enhancing the prediction of treatment outcomes. Furthermore, because surgical resection was found to be an independent factor for both OS and PFS, the PILE score might aid in surgical decision-making, especially for patients who are borderline operable or have high systemic inflammation, by pinpointing those who might benefit from prehabilitation or increased monitoring. Nevertheless, as this study was conducted at a single institution using a specific weekly paclitaxel–carboplatin regimen, wider external validation is crucial. Future multicenter prospective studies across various geographic areas and treatment protocols, including different chemoradiotherapy regimens and combinations with immunotherapy, are necessary to verify the general applicability of the PILE score and define its role in modern ESCC management.

## 5. Conclusions

In summary, the PILE score is a straightforward, accessible, and efficient prognostic tool for forecasting both OS and PFS in patients with locally advanced esophageal squamous cell carcinoma undergoing neoadjuvant chemoradiotherapy. Notably, the prognostic performance of the PILE-based model was stronger for PFS than for OS, as reflected by its superior C-index, indicating better discriminatory ability for early disease progression. Incorporating essential inflammatory and performance metrics offers a thorough evaluation of the patient’s risk. We suggest that these findings be validated in larger multicenter prospective studies to further confirm the significance of the PILE score in guiding risk stratification and personalized treatment plans for patients with ESCC.

## Figures and Tables

**Table 1:** Patient characteristics and laboratory findings.

Variable	*n* (%)/Mean ± SD
Age (years), mean ± SD	62.30 ± 10.84
Sex	
Male	39 (36.1%)
Female	69 (63.9%)
Smoking status	
Non-smoker	72 (66.7%)
Active smoker	32 (29.6%)
Former smoker	4 (3.7%)
ECOG performance status	
0	28 (25.9%)
1	58 (53.7%)
2	22 (20.4%)
Comorbidity	
Absent	57 (52.8%)
Present	51 (47.2%)
Comorbidity types	
None	57 (52.8%)
DM	10 (9.3%)
HT	22 (20.4%)
CAD	11 (10.2%)
COPD	3 (2.8%)
Other	5 (4.6%)
Pathological type	
Squamous cell carcinoma	108 (100%)
Tumor location	
Middle third	27 (25.0%)
Lower third	81 (75.0%)
Clinical T stage	
T2	40 (37.0%)
T3	68 (63.0%)
Clinical N stage	
N0	26 (24.1%)
N1	35 (32.4%)
N2	35 (32.4%)
N3	12 (11.1%)
Clinical stage	
Stage II	40 (37.0%)
Stage III	56 (51.9%)
Stage IVA	12 (11.1%)
Surgery performed	34 (31.5%)
pCR	25 (23%)
Progression	43 (39.8%)
Distant metastasis	14 (13.0%)
Lung	7 (50.0%)
Liver	3 (21.4%)
Other	4 (28.6%)
Status	
Alive	62 (57.4%)
Deceased	46 (42.6%)
Laboratory parameters (mean ± SD)	
LDH (U/L)	216.83 ± 51.55
Lymphocyte count (×10^9^/L)	2.176 ± 0.680
Neutrophil count (×10^9^/L)	5.348 ± 2.013
Monocyte count (×10^9^/L)	0.612 ± 0.195
Platelet count (×10^9^/L)	296.42 ± 101.13
PIV	550.05 ± 577.63
NLR	2.75 ± 1.68
PLR	148.4 ± 66.9
OS (months)	26.34 ± 15.32
PFS (months)	22.01 ± 15.39
PILE score	
0	34 (31.5%)
1	42 (38.9%)
2	26 (24.1%)
3	6 (5.6%)
PILE risk classification	
Low (0–1)	76 (70.4%)
High (2–3)	32 (29.6%)

Abbreviations: CAD = Coronary artery disease; COPD = Chronic obstructive pulmonary disease; DM = Diabetes mellitus; ECOG = Eastern Cooperative Oncology Group; HT = Hypertension; LDH = Lactate dehydrogenase; NLR = Neutrophil-to-lymphocyte ratio; OS = Overall survival; pCR = Pathological complete response; PFS = Progression-free survival; PILE = Composite inflammation–laboratory evaluation score derived from PIV, LDH, and ECOG; PIV = Pan-Immune-Inflammation Value; PLR = Platelet-to-lymphocyte ratio; SD = Standard deviation.

**Table 2:** Univariate and multivariate cox regression analysis for OS.

Variable	Univariate HR (95% CI)	*p*-Value	Multivariate HR (95% CI)	*p*-Value
Age (years)	1.032 (1.005–1.060)	0.021	1.018 (0.984–1.053)	0.312
Sex	0.748 (0.413–1.354)	0.338	0.696 (0.366–1.323)	0.269
Comorbidity	1.242 (0.695–2.219)	0.464	0.834 (0.406–1.715)	0.623
Clinical stage	—	0.111	—	0.514
Stage III vs. II	1.186 (0.608–2.313)	0.616	1.235 (0.608–2.507)	0.559
Stage IVA vs. II	2.377 (1.024–5.518)	0.044	1.706 (0.689–4.227)	0.249
Surgery	0.181 (0.071–0.460)	<0.001	0.249 (0.090–0.683)	0.007
PILE risk (High vs. Low)	3.531 (1.974–6.318)	<0.001	2.527 (1.380–4.629)	0.003

Abbreviations: CI = Confidence interval; HR = Hazard ratio; OS = Overall survival; PILE = Composite inflammation–laboratory evaluation score derived from PIV, LDH, and ECOG.

**Table 3:** Univariate and multivariate cox regression analysis for PFS.

Variable	Univariate HR (95% CI)	*p*-Value	Multivariate HR (95% CI)	*p*-Value
Age (years)	1.030 (1.002–1.060)	0.035	0.986 (0.952–1.022)	0.447
Sex	1.366 (0.701–2.663)	0.359	1.343 (0.649–2.778)	0.427
Comorbidity	1.938 (1.049–3.579)	0.034	1.615 (0.780–3.345)	0.197
Clinical stage	—	0.312	—	0.439
Stage III vs. II	1.162 (0.594–2.272)	0.661	1.596 (0.780–3.262)	0.2
Stage IVA vs. II	1.989 (0.801–4.938)	0.138	1.285 (0.475–3.473)	0.621
Surgery	0.116 (0.041–0.328)	<0.001	0.131 (0.044–0.394)	<0.001
PILE risk (High vs. Low)	3.561 (1.936–6.551)	<0.001	2.932 (1.525–5.639)	0.001

Abbreviations: CI = Confidence interval; HR = Hazard ratio; PFS = Progression-free survival; PILE = Composite inflammation–laboratory evaluation score derived from PIV, LDH, and ECOG.

## Data Availability

The datasets generated and/or analyzed during the current study are not publicly available because of patient privacy and institutional data protection policies but are available from the corresponding author upon reasonable requests.

## Abbreviations

The following abbreviations are used in this manuscript.

AUC	Area Under the Curve
CAD	Coronary Artery Disease
C-index	Concordance Index
CALLY Index	CRP–Albumin–Lymphocyte Index
CI	Confidence Interval
COPD	Chronic Obstructive Pulmonary Disease
dCRT	Definitive Chemoradiotherapy
DM	Diabetes Mellitus
ECOG	Eastern Cooperative Oncology Group
ESCC	Esophageal Squamous Cell Carcinoma
GRIm Score	Gustave Roussy Immune Score
Gy	Gray
HR	Hazard Ratio
HT	Hypertension
LDH	Lactate Dehydrogenase
nCRT	Neoadjuvant Chemoradiotherapy
NLR	Neutrophil-to-Lymphocyte Ratio
OS	Overall Survival
pCR	Pathological complete response
PFS	Progression-Free Survival
PILE	Composite inflammation–laboratory evaluation score derived from PIV, LDH, and ECOG
PIV	Pan-Immune-Inflammation Value
PLR	Platelet-to-Lymphocyte Ratio
R0	Microscopically margin-negative resection
ROC	Receiver Operating Characteristic
SD	Standard Deviation
ULN	Upper Limit of Normal
